# Long-term outcomes of Gamma Knife radiosurgery for trigeminal neuralgia patients with or without concomitant continuous pain

**DOI:** 10.1097/MD.0000000000041026

**Published:** 2024-12-20

**Authors:** Guijiang Dong, Quanqing Li, Jin Sun, E. Chen, Xiaoning Lin, Junjiang Tong, Hongjin Chen, Xiang Yao, Hongbo Wang, Xinhua Tian

**Affiliations:** a Department of neurosurgery, Zhongshan Hospital of Xiamen University, School of Medicine, Xiamen University, Xiamen, China.

**Keywords:** concomitant continuous pain, Gamma Knife radiosurgery, trigeminal neuralgia

## Abstract

The effectiveness of Gamma Knife radiosurgery (GKRS) in treating trigeminal neuralgia (TN) has been demonstrated by a number of previous studies. However, there is a lack of research specifically documenting the initial and long-term outcomes of paroxysmal and persistent pain respectively following GKRS for TN with concomitant continuous pain (CCP). This study retrospectively analyzed pain outcomes and complications in 46 TN patients with CCP and 112 patients without CCP who underwent GKRS as initial surgical intervention at our institution from January 2019 to January 2024. Pain outcomes were classified as excellent (BNI I), good (BNI II–IIIa), and poor (BNI IIIb–V). Demographic and clinical data, pain outcomes, and complications were compared between patients with and without CCP. Subsequently, risk factors for poor outcomes after GKRS were evaluated using univariate and multivariate Cox regression analysis. The initial rate of poor outcomes in TN patients with CCP was similar to that of patients without CCP (15.8% vs 14.4%, *P* = .878). Following a minimum 6-month follow-up, the rate of poor pain outcomes increased to 37.0% in patients with CCP, compared to 38.4% in those without CCP (*P* = .968). Notably, the rate of long-term complete pain relief in patients without CCP was significantly higher than in those with CCP (35.7% vs 15.2%, *P* < .001). Poor response to medication (*P* < .001) was identified as an independent risk factors for poor outcomes after GKRS. While most TN patients with or without CCP can achieve favorable pain outcomes after GKRS, individuals with CCP were less likely to achieve complete pain relief compared to those without CCP.

## 1. Introduction

The main clinical characteristic of trigeminal neuralgia (TN) is paroxysmal, electric shock-like, and unilateral pain in the sensory distribution of the trigeminal nerve.^[1]^ However, a significant proportion of TN patients (20% to 40%) may also experience atypical symptoms.^[2,3]^ This atypical pain is often described as persistent facial pain occurring between attacks within the affected trigeminal region, known as concomitant continuous pain (CCP) as per the 3rd edition of the International Classification of Headache Disorders (ICHD-3), previously referred to as “atypical TN” or “TN type 2.”^[4]^ The enduring, sharp facial pain associated with CCP significantly impacts patients’ quality of life and can lead to psychological issues like anxiety and depression, prompting researchers and neurosurgeons to delve deeper into understanding the etiology and management of this condition.^[5,6]^ Unfortunately, the underlying cause of CCP remains unclear, presenting a considerable challenge in determining effective TN treatments.^[7,8]^

Common surgical methods for treating TN include microvascular decompression (MVD), percutaneous ablative procedures, and Gamma Knife radiosurgery (GKRS).^[9–11]^ Each procedure has its own advantages and limitations, and is tailored to patients with specific clinical characteristics and needs. GKRS is a relatively noninvasive and simpler procedure, and works by blocking overactive nerve conduction in the REZ or retro-Gasserian area.^[12,13]^ Due to its ability to be performed under local anesthesia, GKRS is commonly recommended as the first-line treatment for TN patients with poor general health who are averse to surgical anesthesia, even though MVD has higher efficacy and fewer complications.^[14,15]^ Therefore, GKRS plays a significant role in the surgical management of TN.

Since Lars Leksell pioneering radiosurgical treatment for TN in 1953, numerous studies have highlighted the effectiveness of GKRS for this condition.^[16,17]^ Approximately 24% to 71% of patients experience sustained pain relief 1 to 2 years after GKRS, with 33% to 56% still reporting pain relief at 4 to 5 years.^[18,19]^ However, recent research has identified atypical features as a significant predictor of poor response to GKRS, posing a challenge for clinicians in deciding whether to proceed with this treatment for TN with CCP.^[20]^ Unfortunately, there is a limited number of studies with large sample sizes focusing on the long-term outcomes of GKRS for TN with CCP, leading to inconclusive results. In this study, we collected clinical data from 2 patient cohorts who underwent GKRS at our institution and followed them up for a minimum of 6 months to evaluate the response of TN patients with CCP to GKRS and identify potential risk factors for unfavorable outcomes.

## 2. Patients and methods

### 2.1. Patient characteristic

**Figure 1.**
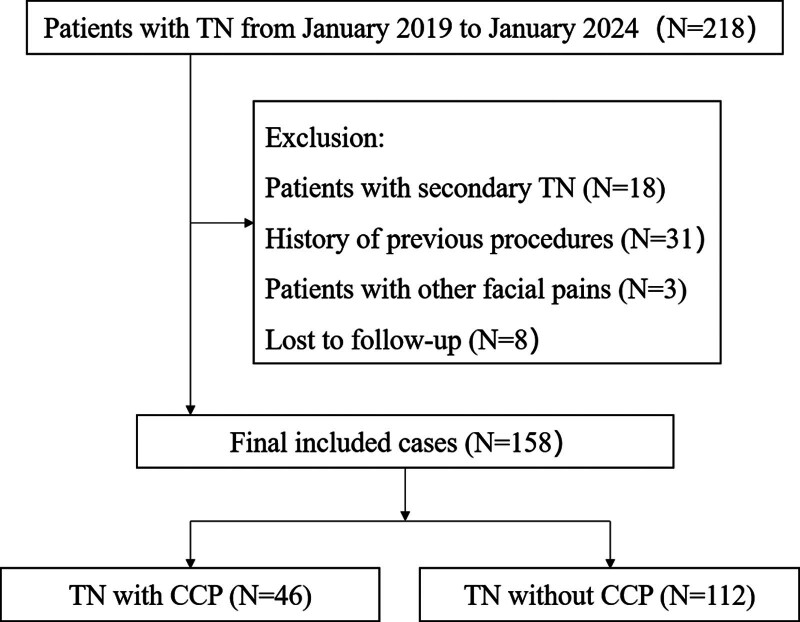
Flowchart of patient screening and grouping.

A total of 218 patients with TN were screened in our department between January 2019 and January 2024. Inclusion criteria encompassed patients with classical or idiopathic TN. Exclusion criteria excluded patients with secondary TN, other types of facial pain, those who had a history of previous MVD, GKRS, or percutaneous surgeries and those who were lost to follow-up or had <6 months of follow-up. The decision to proceed with GKRS was based on preoperative magnetic resonance tomographic nerve angiography images, patient preferences, and overall health status. The screening process for TN patients is illustrated in Figure 1. Finally, 46 TN patients with CCP and 112 TN patients without CCP were included in this clinical study. Demographic and clinical data such as age, side of symptoms, pain duration, pain distribution, neurovascular compression (NVC) on magnetic resonance images, response to carbamazepine or oxcarbazepine, and length of hospital stay for both groups were extracted from the patients’ medical records and are summarized in Table 1. The study was conducted in accordance with the Declaration of Helsinki, and approved by the Institutional Review Board (or Ethics Committee) of Zhongshan Hospital of Xiamen University, School of Medicine, Xiamen University (XMZSYYKY ER No. 2019028).

**Table 1:** Comparison of demographic and clinical characteristics of between TN patients with and without CCP.

Characteristic	TN without CCP (n = 112)	TN with CCP (n = 46)	*P* value
Mean age in yr	64.9 ± 11.3 (38–84)	64.4 ± 11.1 (30–86)	.801
Mean follow-up in months	39.0 (6–66)	35.4 (6–64)	.268
Female (%)	69 (61.6%)	31 (67.4%)	.746
Right (%)	75 (67.0%)	30 (65.2%)	.156
Mean disease duration in months	66.4 ± 64.6 (6–360)	70.2 ± 69.8 (3–360)	.738
Involvement of trigeminal divisions			
V_1_	7 (6.3%)	3 (6.5%)	.863
V_2_	25 (22.3%)	12 (26.1%)	
V_3_	40 (35.7%)	16 (34.8%)	
V_1,2_	9 (8.0%)	3 (10.9%)	
V_2,3_	27 (24.1%)	9 (19.6%)	
V_1,2,3_	4 (3.6%)	1 (2.2%)	
Response to medication			
Yes	102 (91.1%)	30 (65.2%)	.015*
No	10 (8.9%)	16 (34.8%)	
Presence of trigger point			
Yes	92 (83.9%)	31 (67.4%)	.023*
No	18 (16.1%)	15 (32.6%)	
Presence of NVC			
Yes	89 (76.8%)	33 (73.9%)	.450
No	26 (23.2%)	13 (28.3%)	
Presence of facial numbness			
Yes	4 (3.6%)	6 (13.0%)	.026*
No	108 (96.4%)	40 (87.0%)	

Values are expressed as number (%) or as mean ± SD.

Abbreviations: CCP = concomitant continuous pain, NVC = neurovascular compression, TN = trigeminal neuralgia.

* *P* <.05.

### 2.2. Procedure description

The Leksell G Frame (Elekta Instrument AB) was affixed to the patient’s head under local anesthesia. High-resolution (1 mm) stereotactic MRI scans were conducted, including a 3D-TOF-MRA sequence and a T2-weighted sequence (CISS/FIESTA) without contrast. A single 4-mm isocenter was positioned on the trigeminal nerve at the root entry zone (REZ). A dose of 30 to 45 Gy was prescribed to the 50% isodose line for each REZ, with a central radiation dose of 70 to 90 Gy delivered to the planned point of the trigeminal nerve. The radiosurgery plans were approved by a multidisciplinary team including a neurosurgeon, a radiation oncologist, and a medical physicist. Following the procedure, the Leksell frame was removed and sterile dressings were applied to the pin sites before discharging the patients home on the first postoperative day.

### 2.3. Clinical follow-up and outcome assessment

An independent neurosurgeon, uninvolved in patient management during the study period, conducted follow-up assessments with patients over the phone to mitigate selection bias. Patients were asked to report if their facial pain had disappeared, note any pain recurrences, and report any complications. Follow-up duration ranged from 6 to 66 months. Pain intensity and numbness were evaluated using the Barrow Neurological Institute (BNI) pain and numbness intensity scales, respectively (Tables 2 and 3). Pain outcomes were categorized as excellent (BNI I), good (BNI II or IIIa), and poor (BNI IIIb–V). Long-term outcome was determined at the final follow-up. Recurrence was defined as the return of pain following initial improvement, with an intensity of BNI IIIb to V. Bothersome facial numbness was defined by a BNI numbness scale of III to IV.

**Table 2:** Barrow neurological institute facial pain intensity scale.

Grade	Description
I	No pain, no medication
II	Occasional pain, no medication
III_a_	No pain, continue medication
III_b_	Persistent pain, controlled with medication
IV	Some pain, not controlled with medication
V	Severe pain or no relief

**Table 3:** Barrow neurological institute facial numbness intensity scale.

Grade	Description
I	No numbness
II	Mild numbness that is not bothersome
III	Somewhat bothersome numbness
IV	Very bothersome numbness

### 2.4. Statistical analysis

Statistical analysis was conducted using SPSS software version 27.0 (SPSS, Inc., Chicago). Group comparisons were made using either an independent samples *t*-test or Wilcoxon rank-sum test for continuous data, and Pearson chi-square test or Fisher exact test for categorized data as appropriate. Kaplan–Meier survival analyses were used to plot the duration from surgery to a poor outcome (ineffective or recurrence) for different patient cohorts. Factors predicting outcomes were identified through univariate analyses, followed by a multivariate Cox proportional hazards model to assess variables in pain relief. Results were reported as hazard ratios (HRs), corresponding 95% confidence intervals (95% CIs), and respective *P*-values. Statistical significance was set at the .05 level.

## 3. Results

### 3.1. Patient characteristics

During the follow-up, 8 patients were lost to contact, resulting in a 95.2% response rate. This study included a total of 158 patients with TN, with 29.1% having CCP and 70.9% without CCP. Demographic and clinical preoperative characteristics of the 2 patient cohorts are compared in Table 1. Among the 46 patients with CCP, 56.5% experienced continuous pain at disease onset, while 43.5% developed CCP after an average of 16.8 months (range 3–60 months). There were no significant differences between the 2 groups in terms of sex, age, laterality, disease duration, trigeminal division involvement, or presence of neurovascular compression. The onset age of TN was predominantly over 50 years, with pain more common in females and on the right side in both groups. Predominantly, V2 and V3 were affected, and the distribution of CCP aligned with the area of paroxysmal pain. Pain duration ranged from 6 to 360 months (mean 66.4 months) in patients without CCP and 3 to 360 months (mean 70.2 months) in patients with CCP. Notably, there were significant differences in medication response, presence of trigger points, and sensory abnormalities between the groups. TN patients without CCP had a better response to medications compared to those with CCP (66.7% vs 83.1%, *P* = .015). The incidence of preoperative facial numbness was significantly higher in patients with CCP compared to those without CCP (13.0% vs 3.6%, *P* = .026), while the presence of trigger points was significantly lower (67.4% vs 83.9%, *P* = .023).

### 3.2. Pain outcomes of TN patients with or without CCP

**Figure 2.**
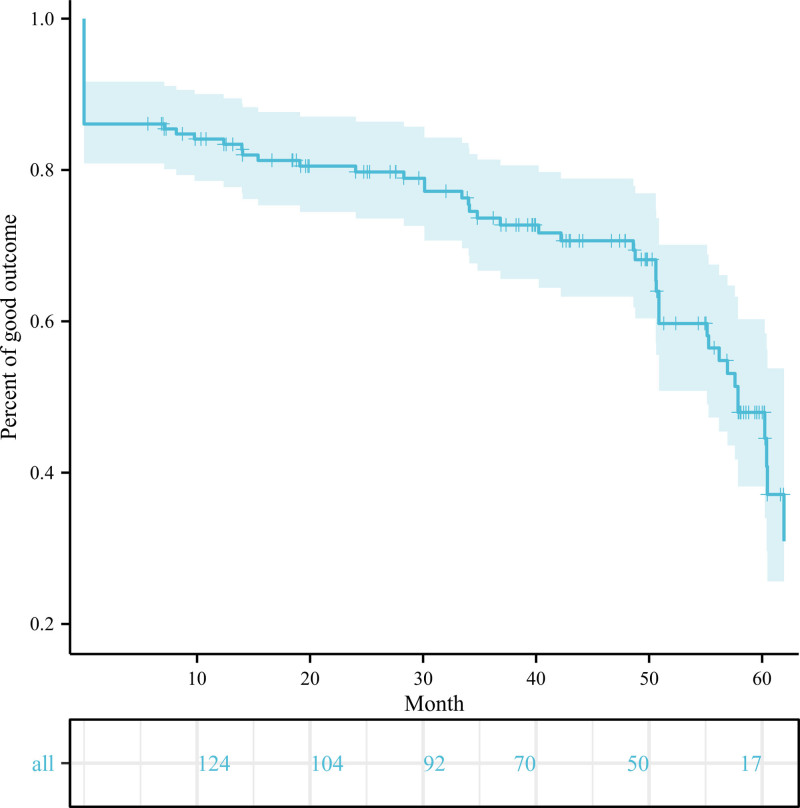
Illustration of pain recurrence following Gamma Knife radiosurgery for patients with TN over time. TN = trigeminal neuralgia.

The mean follow-up period was 39.0 months (range, 6–66 months) for patients with CCP and 35.4 months (range, 6–64 months) for patients without CCP. Following GKRS, 86.1% of TN patients reported excellent or good outcomes (BNI I, 50.6%; BNI II–IIIa, 35.4%) within 1 to 24 weeks. However, 13.9% of patients did not experience significant pain relief. At the final follow-up, only 62.0% of TN patients reported significant pain relief (BNI I, 31.6%; BNI II–IIIa, 30.4%). The Kaplan–Meier plot depicting the pain recurrence after GKRS for all patients over time is illustrated in Figure 2.

**Figure 3.**
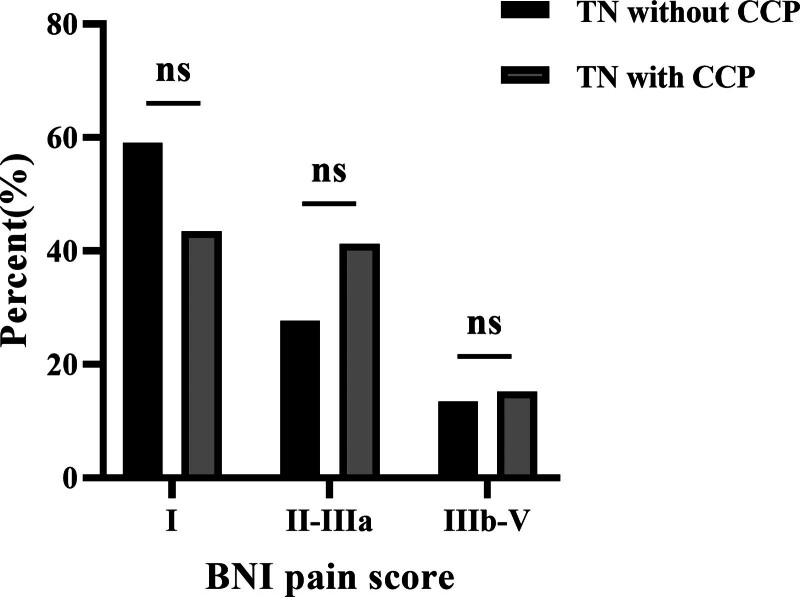
Initial BNI pain scores in patients with and without concomitant continuous pain following Gamma Knife radiosurgery. BNI = barrow neurological institute, Ns = no significant difference.

**Figure 4.**
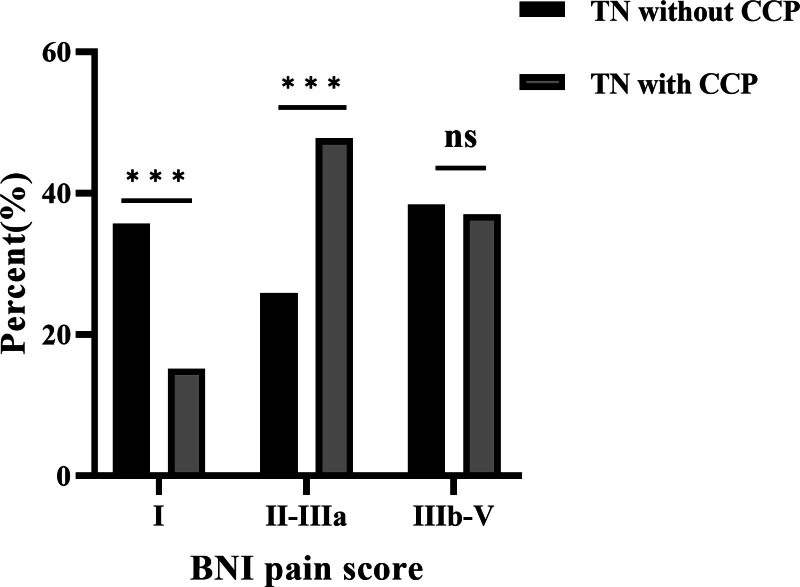
Long-term BNI pain scores in patients with and without concomitant continuous pain following Gamma Knife radiosurgery. ***: *P* < .001. BNI = barrow neurological institute.

**Figure 5.**
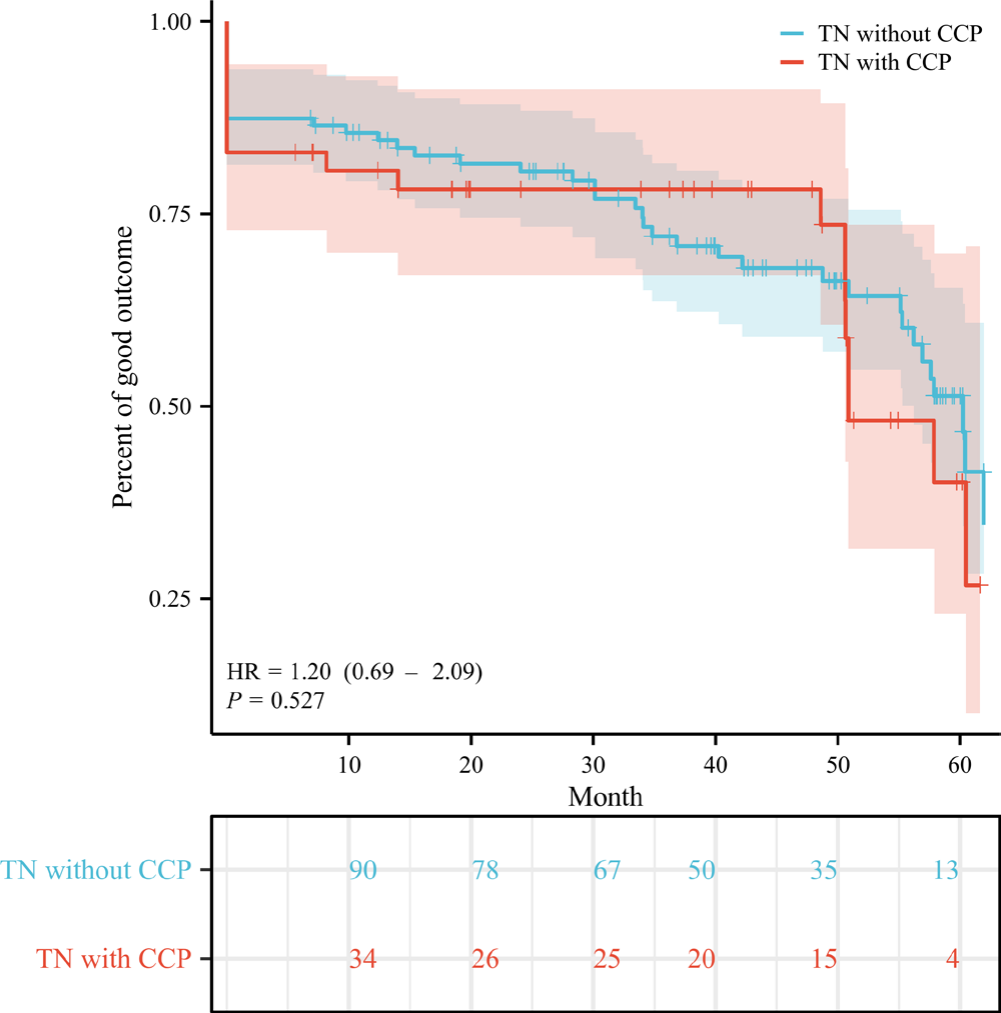
Kaplan–Meier survival curves for patients with and without concomitant continuous pain. CCP = concomitant continuous pain, HR = hazard ratio, TN = trigeminal neuralgia.

When comparing pain outcomes between patients with and without CCP, no significant differences were observed in both initial (15.2% vs 13.4%, *P* = .763) and long-term (37.0% vs 38.4%, *P* = .838) poor outcome rates. The initial and long-term BNI pain scores were compared between the 2 groups, as shown in Figures 3 and 4, respectively. The Kaplan–Meier plots for both patient cohorts were highly similar (χ^2^ = 0.128, *P* = .721) (Fig. 5). The mean time from GKRS to pain relief were similar between patients with and without CCP (4.1 vs 5.3 weeks, *P* = .063). 10 patients with CCP (mean 22.5 months, range 9–48 months) and 28 patients without CCP experienced pain recurrence (mean 21.4 months, range 6–48 months) during the follow-up period. The difference in time-to-recurrence between the groups was not statistically significant (*P* = .836). Besides, it was found that the rate of long-term excellent outcomes in patients without CCP was significantly higher than in those with CCP (35.7% vs 15.2%, *P* < .001). However, the rates of initial excellent outcomes did not show a significant difference between the 2 groups (59.0% vs 43.5%, *P* = .076).

### 3.3. Outcomes of paroxysmal pain and CCP in TN patients with CCP

For TN patients with CCP, the study recorded and compared the initial and long-term BNI scores of paroxysmal pain and CCP separately (Table 4). While the rates of poor outcomes for both initial and long-term paroxysmal pain were lower than those of CCP, the differences were not statistically significant (*P* = .536, *P* = .508). It is worth noting that the rate of long-term excellent outcomes for paroxysmal pain was significantly higher than that for CCP, with 39.1% achieving excellent outcomes compared to 15.2% for CCP (*P* = .017).

**Table 4:** Initial and long-term paroxysmal and concomitant continuous pain outcomes after GKRS.

	Paroxysmal pain	CCP	*P*-value
Initial BNI score			
I	22 (47.8%)	14 (30.4%)	.231
II to IIIa	19 (41.3%)	25 (54.3%)	
IIIb to V	5 (10.9%)	7 (15.2%)	
Long-term BNI score			
I	18 (39.1%)	7 (15.2%)	.017*
II to IIIa	14 (30.4%)	22 (47.8%)	
IIIb to V	14 (30.4%)	17 (37.0%)	

Values are expressed as number (%).

Abbreviations: CCP = concomitant continuous pain, GKRS = Gamma Knife radiosurgery.

* *P*<.05.

### 3.4. Preoperative risk factors for poor outcomes following GKRS

**Figure 6.**
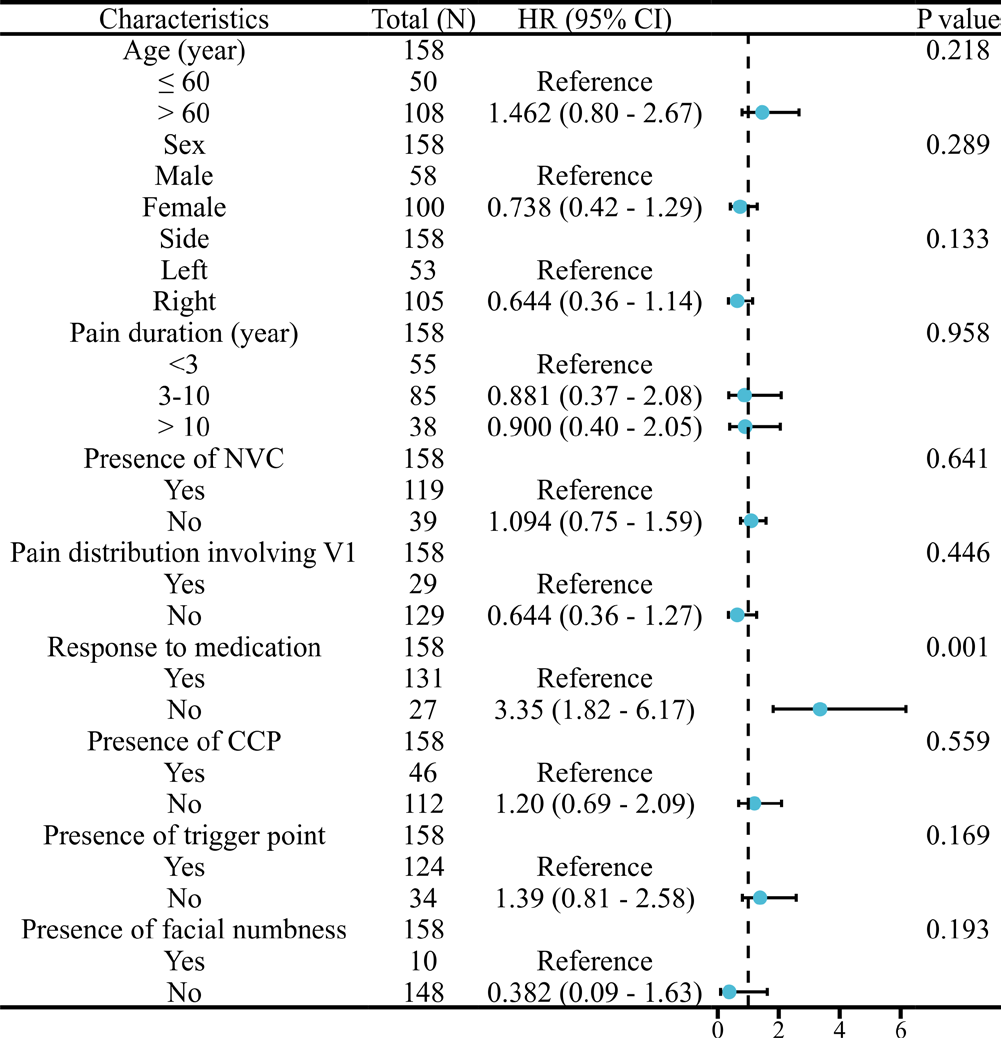
Forest plot of the univariable Cox regression analysis of clinical characteristics. CCP = concomitant continuous pain, HR = hazard ratio, NVC = neurovascular compression.

**Figure 7.**
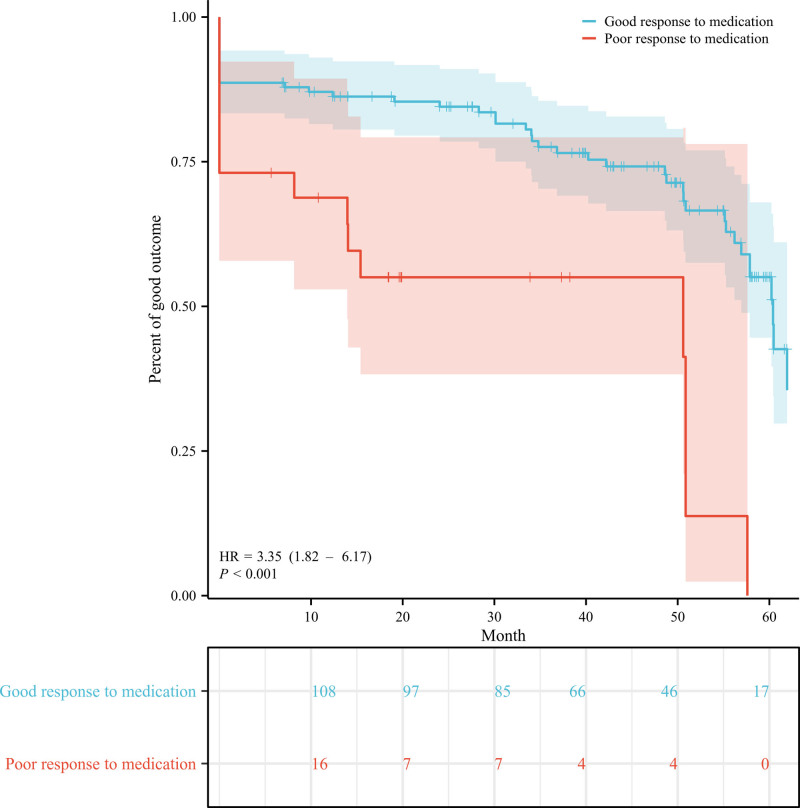
Kaplan–Meier survival curves for patients with good and poor response to medication. HR = hazard ratio.

Overall preoperative predictors of poor outcomes following GKRS were analyzed and demonstrated in Figure 6. Through univariate analysis, only 1 variable was identified as being associated with poor outcomes after GKRS: poor response to medications (HR 3.35, 95% CI: 1.82–6.17, *P* < .001). Kaplan–Meier plot for patients with different response to medication is displayed in Figure 7. Subsequently, a multivariate stepwise selection Cox hazards analysis revealed that poor response to medications (HR 2.90, 95% CI: 1.76–5.96, *P* < .001) was an independent risk factor for poor outcomes following GKRS.

### 3.5. Complications

Among patients with CCP, 20 individuals (43.5%) experienced postoperative facial numbness. Out of these, 16 (34.8%) were classified as grade II (mild facial numbness, not bothersome), while 4 (8.7%) were classified as grade III/IV (somewhat bothersome/very bothersome). In contrast, in patients without CCP, there were 70 individuals (62.5%) classified as grade I (no facial numbness), 28 (25.0%) as grade II, and 14 (12.5%) as grade III/IV. Statistical analysis showed no significant difference between the groups (*P* = .442).

## 4. Discussion

There is currently no consensus on the most effective treatment approach for TN with CCP, and only a few studies have focused on the outcomes of paroxysmal and persistent pain following GKRS for TN with CCP. This study examines the long-term surgical outcomes of TN patients with and without CCP who underwent GKRS at a single institution. The key finding indicates that most TN patients, with or without CCP, can experience significant pain relief after GKRS, but individuals with CCP are less likely to achieve complete pain relief compared to those without CCP.

### 4.1. Clinical characteristics of TN patients with CCP

In this clinical study, TN with CCP accounted for 29.1% of all cases, a proportion consistent with a previous study by Li et al involving 175 patients, where 67.4% had purely paroxysmal pain and 32.6% had CCP.^[21]^ Interestingly, not all CCP occurred with paroxysmal pain at the same time. Among the cohorts studied, only 56.5% experienced continuous pain at disease onset, while the remaining 43.5% developed this condition on average 16.8 months (range 3–60 months) after experiencing paroxysmal pain. Therefore, it was proposed that CCP may evolve from paroxysmal pain through central sensitization mechanisms and TN patients should accept surgical treatments in the early stages of the disease.^[22]^

When comparing the baseline characteristics between TN patients with and without CCP, no significant differences in terms of age, gender, laterality, disease duration, or trigeminal division involvement were observed. Several researchers have noted that the nature of paroxysmal pain and CCP are distinct, and NVC may not be the contributing or main cause of CCP.^[23]^ However, the incidence of NVC was similar between different patient cohorts. One study suggested that CCP occurrence was linked to trigeminal root atrophy (NVC degree) rather than NVC incidence, site, or dislocation.^[24]^ Hence, the association between CCP and NVC remains unclear. In addition, we found that patients with CCP showed poorer response to medication compared to those without CCP (66.7% vs 83.1%, *P* = .015). Currently, there is no highly effective drug for treating CCP, and even carbamazepine and oxcarbazepine, effective in typical TN patients, have limited analgesic effects on CCP.^[25,26]^ Li et al study demonstrated that medications targeting neuropathic pain may be more beneficial for CCP, but stronger evidence is needed to support this claim, highlighting the necessity for further research.^[23,27,28]^ Facial numbness is a common post-surgery complication, but preoperative facial numbness is not prevalent in either patient cohort. Interestingly, patients with CCP are more likely to experience preoperative facial numbness (13.0% vs 3.6%, *P* = .026). Li et al hypothesized that a narrow foramen ovale may trap the mandibular nerve and lead to CCP, providing a potential explanation for this phenomenon.^[27]^

### 4.2. Outcomes of pain in patients with or without CCP

Numerous previous studies have reported their experience treating TN with GKRS.^[29,30]^ In our study, Following GKRS, 86.1% of TN patients reported excellent or good outcomes (BNI I, 50.6%; BNI II–IIIa, 35.4%) within 1 to 24 weeks. However, 13.9% of patients did not experience significant pain relief. During follow-up, 27 patients suffered from pain recurrence 6 to 48 months following GKRS, with only 62.0% of TN patients experiencing significant pain relief (BNI I, 31.6%; BNI II–IIIa, 30.4%). The proportion of initial pain relief was comparable to previous studies on GKRS, with initial pain relief rates ranging from 83.0% to 94.8%.^[12,31,32]^ However, the long-term pain outcome was slightly poorer compared to the previous reports, which may be due to strict good outcome assessment criterion.

While there is a wealth of literature on GKRS for TN, there has been limited focus on its efficacy for TN with CCP. Previous studies have examined the impact of GKRS on TN with CCP or atypical pain, but many of these cases involved patients with a history of prior procedures. This history could potentially alter the nature of paroxysmal pain to trigeminal deafferentation pain, affecting the classification of TN.^[13,33]^ Furthermore, no study has specifically documented the paroxysmal and persistent outcomes of TN patients with CCP following GKRS. Therefore, our research aims to provide insights for treating TN patients considering GKRS as their initial procedure.

Although the presence of CCP has been viewed as a potential risk factor for unfavorable outcomes after surgical procedures, previous studies have shown a high rate of pain relief and no significant difference in pain outcomes between patients with and without CCP. Dhople et al found that patients with atypical TN could experience comparable rates of pain relief to those with classical TN (72% vs 81%, *P* = .304).^[13]^ In this study, GKRS resulted in excellent and good pain outcomes in 43.5% and 84.8% of patients with CCP, compared with 59.0% and 86.6% in those without CCP initially. After a minimum 6-month follow-up, the excellent and good pain outcomes rates decreased to 15.2% and 63.0% in patients with CCP, compared with 35.7% and 61.6% in those without CCP. The differences in the initial and long-term good pain outcomes rates between patients with and without CCP were not statistically significant (*P* = .878, *P* = .968, respectively). The pain relief rates and comparison results were consistent with previous literature.^[34]^ Interesting, it was noted that TN patients with CCP were more difficult to achieve complete pain relief (BNI I) compared to those without CCP, while the significant relief rates were similar. Moreover, the excellent outcome rate of TN patients with CCP after GKRS was lower than that after CyberKnife, MVD and percutaneous procedures.^[21,33,35–37]^ Consequently, for patients with CCP who expect to get complete pain relief, GKRS may not be the most suitable choice and other procedures should be recommended.

Since TN with CCP present with 2 kinds of pain with distinct nature and characteristics, we exanimated both of them respectively. It is important to note that while CCP can be present at onset, the majority (72%) of cases begin after the occurrence of paroxysmal pain.^[2]^ Researchers have hypothesized that CCP may be caused by central sensitization or by a narrow foramen rotundum and foramen ovale; however, the exact pathogenesis remains unknown.^[22,23]^ As a result, current treatments for CCP are primarily based on clinical experience rather than a clear understanding of the underlying causes. Unfortunately, we were unable to determine whether the timing of the onset of CCP affects its response to GKRS due to incomplete patient data in this study. The initial proportions of excellent and good outcomes in patients with CCP were slightly lower than those without CCP with no significant difference noted. However, the long-term excellent outcome rate of CCP was significantly lower than that of paroxysmal pain in both patients with and without CCP. Additionally, the outcome of paroxysmal pain between patients with and without CCP was not statistically different. Consequently, low complete pain relief in TN with CCP was mainly attributed to CCP instead of paroxysmal pain. The difference in complete pain relief rate between CCP and paroxysmal pain following GKRS may reflect that the pathogenesis of paroxysmal pain and CCP is distinct. Our findings were also observed in other treatment modalities for TN. Li et al reported that CCP persisted in several patients with TN, although paroxysmal pain was significantly relieved following percutaneous balloon compression.^[21]^ The phenomenon of paroxysmal pain and CCP exhibiting varying responses to the same treatment modality remains insufficiently understood and requires further investigation.

### 4.3. Risk factors associated with poor outcome

To further identify the potential predictive factors of poor outcomes following GKRS for TN, a multivariate Cox was performed and revealed that poor response to medications (HR 2.90, 95% CI: 1.76–25.96, *P* < .001) was an independent risk factor for poor outcomes after GKRS. Importantly. poor response to medications has also been found to be associated with poor MVD and percutaneous procedures outcomes in other studies, thus posing a significant challenge on how to improve prognosis of TN with CCP. In addition to response to medication, factors such as typical form of TN, no previous microvascular decompression and Seniority > 60 years were also found to be significantly associated with pain-free outcome after GKRS.^[20,38,39]^ However, these factors were inconsistent among studies and current predictive models seldom were applied in clinic. The difference in the predictive factors between studies may be due to different inclusion and exclusion criteria, Gamma Knife dose and target and outcome assessment.

### 4.4. Complications

GKRS is considered to be relatively less invasive compared to MVD, however, the rate of postoperative facial numbness is higher. In all cases, no severe complications were reported, but 43.5% of individuals with CCP and 37.5% without CCP experienced varying degrees of postoperative facial numbness. Only 8.7% of patients with CCP and 12.5% without CCP described facial numbness as bothersome (BNI numbness III/IV). Our results indicate that GKRS is a safe surgical method for both patients with and without CCP, which is consistent with previous conclusions.^[13,40]^

### 4.5. Strengths and limitations

Our study presents both strengths and limitations. Unlike previous studies that have included TN patients with atypical pain, our research specifically differentiates CCP from other atypical pain and exclusively focuses on patients with CCP. Additionally, we documented the long-term outcomes of TN patients experiencing both paroxysmal and persistent pains separately. Notably, our population represents the largest cohort analyzing pain outcomes in this specific patient group compared to other similar studies. However, among its limitations, the retrospective nature of our study introduces inherent treatment bias. Furthermore, the mean follow-up time was insufficient to observe certain recurrences. While these limitations may affect the generalizability of our findings, they still provide valuable insights that can inform management decisions for TN patients with CCP.

## 5. Conclusions

GKRS can result in significant relief of both episodic and constant pain for TN with CCP. The presence of CCP should not be considered a contraindication for GKRS. However, it is essential to inform patients with CCP about the comparatively lower likelihood of achieving complete pain relief. Our findings can assist physicians in selecting suitable patients with TN for GKRS.

## Author contributions

**Conceptualization:** Guijiang Dong, Quanqing Li, E Chen, Xiaoning Lin.

**Data curation:** Guijiang Dong, Quanqing Li, Xiaoning Lin, Hongbo Wang.

**Formal analysis:** Guijiang Dong, Quanqing Li, Xiaoning Lin, Hongbo Wang.

**Investigation:** Guijiang Dong, Jin Sun, Junjiang Tong, Hongbo Wang.

**Methodology:** Guijiang Dong, Jin Sun, Junjiang Tong, Xiang Yao.

**Project administration:** Guijiang Dong, E Chen, Xiaoning Lin, Junjiang Tong, Xiang Yao.

**Resources:** E Chen, Junjiang Tong, Hongjin Chen, Xiang Yao.

**Software:** Guijiang Dong, Junjiang Tong, Hongjin Chen, Xiang Yao.

**Supervision:** Hongjin Chen, Xinhua Tian.

**Validation:** Guijiang Dong, Hongjin Chen.

**Visualization:** Guijiang Dong, Hongjin Chen, Xinhua Tian.

**Writing – original draft:** Guijiang Dong.

**Writing – review & editing:** Guijiang Dong, Xinhua Tian.
